# Motility Control of Symbionts and Organelles by the Eukaryotic Cell: The Handling of the Motile Capacity of Individual Parts Forges a Collective Biological Identity

**DOI:** 10.3389/fpsyg.2019.02080

**Published:** 2019-09-10

**Authors:** Guglielmo Militello

**Affiliations:** Department of Logics and Philosophy of Science, IAS-Research Centre, University of the Basque Country, San Sebastián, Spain

**Keywords:** motility, interactive autonomy, constitutive autonomy, eukaryotic cell, collective biological identity, symbionts, mitochondria, plastids

## Abstract

Motility occupies a decisive role in an organism’s ability to autonomously interact with its environment. However, collective biological organizations exhibit individual parts, which have temporally or definitively lost their motor capacities, but still able to autonomously interact with their host. Indeed, although the flagella of bacterial symbionts of eukaryotic cells are usually inhibited or lost, they autonomously modify the environment provided by their host. Furthermore, the eukaryotic organelles of endosymbiotic origin (i.e., mitochondria and plastids) are no longer able to move autonomously; nonetheless, they make a cytoskeletal-driven motion that allows them to communicate with other eukaryotic cells and to perform a considerable number of physiological functions. The purpose of this article is twofold: first, to investigate how changes in the motile capacities of the parts of a nested biological organization affect their *interactive autonomy*; second, to examine how the modification of the interactive autonomy of the individual parts influences the *constitutive autonomy* of the collective association as a whole. The article argues that the emergence and maintenance of collective biological identities involves a strict control of the motile abilities of their constituting members. This entails a restriction, but not necessarily a complete loss, of the agential capacities of the individual parts.

## Introduction

By *collective* (or *nested*) biological organizations, we mean biological entities consisting of different parts, each having their own genetic and phenotypic identity. Symbiotic associations and ecosystems are pre-eminently examples of nested organizations, as the biological members of these associations exhibit distinct genomes and specific phenotypic features. The eukaryotic cell is now a unique functionally integrated individual, but its evolutionary origin dates to two (so far proven) endosymbiotic events: the endosymbiosis between an α-proteobacterium and the proto-eukaryotic cell is at the origin of *mitochondria*, whereas the endosymbiosis between a cyanobacterium and the proto-eukaryotic cell gave rise to *plastids*. Accordingly, eukaryogenesis is currently explained as a progressive transformation of a *nested biological organization* into a *functionally integrated individual* that still saves some traces of its symbiotic past (Martin et al., 2015).

The interaction among the members of a collective association is complex and includes a variety of processes ranging from metabolic fluxes to chemical signals involved in coordinated gene expression. An important, yet neglected, aspect of nested associations is the *motility of their parts*, because the motile capacities of components are severely constrained by the whole association. Since a living being can reach its nutrients in the environment and interact with its surroundings by means of motile capacities, the way in which motility is controlled and constrained affects the biological capacities not only of the parts but also of the collective association as a whole.

This article aims at exploring how the constraints imposed on the motility of the individual parts (i.e., symbionts and organelles) of an eukaryotic cell affect their autonomous interactive capacities and at evaluating how this affects the constitutive autonomy of the overall collective association. Accordingly, the key question of this article can be stated as follows: how can a *collective identity* emerge from the control and transformation of the motility of the individual parts?

In order to address this issue, we will analyze how the motility of the symbionts of the eukaryotic cell is controlled by the host so as to^1^ enable the self-maintenance of the whole symbiotic association. The control of motility occupies a decisive role not only in ongoing symbiotic associations but also in the transformation of endosymbiotic proto-mitochondria and proto-plastids into eukaryotic organelles: indeed, the eukaryotic cytoskeleton tightly controls the movement of eukaryotic organelles in such a way that physiological functions and homeostatic regulatory mechanisms can be performed. Accordingly, from an evolutionary point of view, the eukaryotic cytoskeleton has introduced biological novelties that permitted a proto-eukaryotic cell and its endosymbionts to achieve a functionally integrated individuality.

In the light of the above, the main issue of this article will be explored by addressing the following theoretical questions:

1. How is the motility of symbionts controlled by the host so as to enable the self-maintenance of the overall symbiotic association?
2. How is the motility of eukaryotic organelles controlled by cytoskeleton?
3. What is the role played by the eukaryotic cytoskeleton in controlling the interactive capacities of endosymbionts and organelles and how does it affect the biological identity of the eukaryotic cell?

The analysis of these three questions sheds light on the organizational role played by motility in symbiotic associations as well as in individuals (i.e., the eukaryotic cell) based on the integration of closely related units (i.e., eukaryotic organelles). Furthermore, the different interactive behaviors of symbionts and organelles will shed light on their different organizational roles within the eukaryotic cell and explain why they are differently controlled.

The article is divided as follows: in section “Interactions as the Cornerstone of Symbiotic Associations and Autonomous Organisms”, we present a critical review of the current debate on the individuality of symbiotic associations and some theoretical accounts of the relationship between “interactive” and “constitutive” autonomy. The following two sections will examine the physical constraints acting on the motility of eukaryotic symbionts (section “The Control of Symbiotic Motility”) and eukaryotic organelles (section “Mobility of Eukaryotic Organelles”). Section “Interactive Dynamics and the Organizational Role of the Eukaryotic Cytoskeleton” will explore the role played by the eukaryotic cytoskeleton in the control of motility and the evolutionary innovations that it has introduced. Finally, section “Concluding Remarks: The Relationship Between Motility and Biological Autonomy” makes some concluding remarks concerning the relationship between motility and biological autonomy.

## Interactions as the Cornerstone of Symbiotic Associations and Autonomous Organisms

Over the past years, an increasing number of studies have stressed the cardinal importance of symbiotic interactions for defining a biological individual. The eukaryotic cell, notably in multicellular organizations, forms a nested ecosystem with their bacterial symbionts in such a way that they form a unique collective identity based on their mutual interactions (McFall-Ngai et al., 2013). Although the term “holobiont” currently designates the relationship between a multicellular eukaryote with its bacterial symbionts, Margulis (1993) employed this term to refer to a general symbiotic association between a symbiont and a host. The variety of symbiotic associations is extremely wide, since they range from prokaryote-prokaryote interactions [e.g., the *Candidatus Tremblaya princeps-Candidatus Moranella endobia* consortium of *Planococcus citri* (McCutcheon and von Dohlen, 2011) or the bacterial communities of biofilms (Saxena et al., 2019)], protist-prokaryote relationships [e.g., the *Paulinella chromatophora-cyanobacteria* couple (Bodył et al., 2007)], protist-multicellular eukaryotes relationships [e.g., *Giardia lamblia* and the gut of many mammals (Adam, 2001)], to prokaryotes-multicellular eukaryotes associations [e.g., the bacteria living within human gut (Thursby and Juge, 2017)]. On the basis of the location of the symbiont with respect to the host, we separate ectosymbionts (or epibionts) from endosymbionts (Moya et al., 2008): the former live on the surface of their host, whereas the latter within them.

All the aforementioned symbiotic associations are able to *self-maintain* by means of a number of *constitutive* interactions among symbiotic partners: metabolic, genetic, developmental, and immunological interactions (Moya et al., 2008; Gilbert et al., 2012). Metabolic relationships occur when symbiotic partners interchange a number of metabolites, nutrients, and enzymes in such a way that the host provides the symbiont with the nutrients, and in turn, the symbiont supplies the host with the necessary enzymes for assimilating these nutrients or for synthesizing metabolic components (Moya et al., 2008). Genetic interactions consist of the interchange of genetic material among symbiotic partners; this phenomenon, also called as “horizontal gene transfer” (HGT), favors genetic variability, and it is an important source of phenotypic complexity (Ochman and Moran, 2001; Moran, 2007). The development of many invertebrates and vertebrates is partly dependent on their symbionts, because symbionts may provide larvae or embryos of the host with nutrients in such a way that “development then becomes a matter of interspecies communication” (Gilbert et al., 2012, p. 328). Finally, the immune system of the host provides its symbionts with niches, where they can grow and in turn, symbionts enhance the pathogen immunity of their host (Chiu and Gilbert, 2015; Gilbert and Tauber, 2016).

The capacity of self-maintenance of nested biological organizations needs to be studied in close connection with their ability to interact with the surroundings. Studies on prokaryotic endosymbionts of insects have suggested that these prokaryotes exhibit a highly reduced number of genes for cell motility (Moya et al., 2008; Degnan et al., 2010; Manzano-Marín et al., 2012). This suggests that endosymbiosis and maybe also ectosymbiosis impose some constraints on the motility of the individual parts in such a way that the motility of the symbiont(s) is modified and sometimes restricted. One of the reasons why symbiotic associations (particularly endosymbionts) exhibit different environmental conditions compared to the free-living lifestyle is that the micro-environment provided by the host generates a niche with different conditions of life compared to free-living organisms (Moya et al., 2008).

From a philosophical point of view, it has been emphasized that the autonomy of a biological organization relies on two main dimensions: the *constitutive* aspect and the *interactive* dimension. The former includes all those aspects (e.g., metabolism, regulatory processes, immunology, development, etc.) that contribute to the self-maintenance of an individual. The latter entails the capacities (e.g., perception, motility, and action) that allow an organism to interact with the environment and to change it according to its own internal norms (Moreno and Mossio, 2015; Mossio and Bich, 2017).

The constitutive and the interactive dimension are *mutually dependent*, giving rise to an “organizational closure” in such a way that the environment constrains the internal processes of an agent, and an agent exerts some constraints on its own boundary conditions (Moreno and Mossio, 2015, chap. 4). Indeed, a living being could not undergo metabolic processes, if it had not access to the nutrients that are present in the environment. Therefore, minimal forms of agency are required to allow an organism to reach its nutrients, prey, or escape from its predator. In this respect, we can state that the constitutive dimension requires the interactive one. Nonetheless, the opposite holds true as well: the interactive capacities need not only the energy (in the form of ATP molecules) supplied by metabolic processes but also regulatory mechanisms that adapt agential capacities to the features of the environment. Accordingly, the interactive dimension entails the constitutive one and it could not exist without it.

The concept of *“agency,”* which plays a major role both in life and cognitive sciences, summarizes the main aspects of the *autonomous interactive dimension*. Indeed, an individual is an agent if it exhibits a clear distinction between the interior (e.g., the cellular environment) and the exterior (e.g., the surroundings) (individuality criterion); if it is the source of activity (interactional asymmetry criterion); and if it acts according to its own norms or goals (normativity criterion) (Barandiaran et al., 2009). An agent must be able to *modulate* and *control* its behavior in accordance with environmental circumstances, which, in turn, is possible only if a system “is able to evaluate sequentially temporal situations and determine which possibility is functional at each moment in time. […] Thus, an agent has the ability not just to avoid negative tendencies, but to actively seek to improve its situation” (Moreno, 2018, p. 293). In this sense, agency is a kind of *adaptive behavior* that can be fulfilled by two different types of mechanisms: either by modifying the constitutive organization of the system (i.e., metabolism or development) or by modifying the external conditions of the system (i.e., modification of the environmental conditions of the system). Moreno (2018) proposes a simple but valuable model for explaining an autonomous minimal agent: a system is a minimal agent if it has a regulatory subsystem that *modulates* all those inputs that produce *functional modifications* of the e*nvironmental* conditions. The regulatory subsystem consists of a self-production network (i.e., a metabolic system) and a dynamically decoupled regulatory subsystem exerting control actions (Moreno, 2018, p. 295). Within this theoretical framework, agency is a cyclical process that requires that “the effector processes be modulated in accordance with the detected environmental conditions” (Moreno, 2018, p. 296).

A very important aspect of agency is motility, which is “an agent’s capacity to move under its own power, so that it is able to perform fast (relative to its size) directional movements aimed at changing its environment in search of more favorable conditions” (Moreno and Mossio, 2015, p. 102). Motion favors a specific position of the agent with respect to its surroundings in such a way that “motility-based interaction (i.e., behavior) embeds the agent in an active sensorimotor coupling with the environment” (Arnellos and Moreno, 2015, p. 334). It has been claimed that all agents (from the simplest prokaryotes to the most complex multicellular eukaryotes) exhibit a coupling between sensory inputs (e.g., environmental cues, attractants, or repellents) and motor capacities in such a way that perception and action are inextricably connected (Moreno and Etxeberria, 2005; Moreno and Mossio, 2015; Di Paolo et al., 2017)^2^. Agential behavior is strongly influenced by environmental stimuli and also by size-time limitations^3^ (Moreno and Etxeberria, 2005; Moreno and Mossio, 2015).

To conclude, the concept of “agency” has been studied in free-living organisms in close connection with their sensorimotor abilities. Nevertheless, symbiotic associations pose different constraints on the motility of their individual members in such a way that the *organizational conditions* for *agency* in nested biological associations are distinct from those of free-living organisms. This fundamental aspect of symbiotic interactions will be addressed in the following section.

## The Control of Symbiotic Motility

The interactive dimension of prokaryotes relies on the very efficient motile systems that provide them not only with the essential means of locomotion but also with an important material constraint on metabolism. Indeed, the supply of nutrients is made possible by a specific system that links the picking up of environmental signals of nutrients with locomotion. The locomotion of prokaryotes is performed by three kinds of systems: flagella, type IV pili, and cytoskeletal- and cell surface-based movements (Jarrell and McBride, 2009). Bacterial symbionts of unicellular and multicellular eukaryotes are broadly characterized by the modification of their motility systems, and more globally, interactive capacities. In this section, we examine the role played by motility in the establishment of symbiotic relationships; notably, we focus on three distinct symbiotic processes: biofilms^4^, endosymbionts, and ectosymbionts.

Biofilms are symbiotic communities of single- or multi-species bacteria that arise when they attach to an *abiotic* or *biotic* surface, by means of adhesins, leading to a monolayer or multilayer biofilms (Karatan and Watnik, 2009). The biofilm life cycle is characterized by important changes in the motility of its bacterial components. At the beginning, the attachment of bacteria to a surface is strongly favored by *flagella-mediated motility*, because flagella may facilitate the bacterial attachment to surfaces by overcoming *repulsive forces* at the surface-medium interface. Flagella may also promote the bacterial movement of growing cells along an abiotic surface in such a way that the spread of a biofilm is encouraged (Pratt and Kolter, 1998). The attachment to a surface is also promoted by type IV pili, because they contain a specific adhesin (the mannose-specific adhesin, FimH) that allows a stable cell-to-surface attachment (O’Toole and Kolter, 1998; Pratt and Kolter, 1998).

When the bacterial population increases and overcomes a threshold, the motility of individual bacteria is *inhibited* in order to promote the constitution of the extracellular polymeric substance (EPS) matrix^5^. The reduction of motility is achieved by means of post-translational modifications^6^, transcriptional regulation^7^, and quorum sensing (QS) system^8^ (Guttenplan and Kearns, 2013). During the existence of the EPS matrix, the motility of single bacteria is impeded. However, the EPS matrix is an ephemeral structure that disassembles in response to environmental substances concentration or bacterial lysis. The *re-activation of the genes* responsible for bacterial motility is a crucial aspect of the disassembly of the EPS matrix, and therefore, the destruction of a biofilm and the re-appearance of the planktonic state. Recent studies have shown that the dispersion of a biofilm can be promoted by the synthesis of bacterial flagella (as in *E. coli*) or by the production of mushroom-like pillars of bacteria (as in *P. aeruginosa*) (Karatan and Watnik, 2009).

It is worth stressing that in biofilms, the inhibition of bacterial motility is *not* performed by the host (i.e., the abiotic or biotic surface), but it is rather the outcome of the signals triggered by the EPS matrix. Biofilm is an interesting case of how the *collective control of the motility of parts* allows the emergence of nested biological organization. However, let us focus now on two kinds of symbiotic associations – endosymbiosis and ectosymbiosis – in which the motility of the symbiont is controlled by the *host*.

The *inhibition of motility* is common in bacterial endosymbionts and it is due either to the *loss of the genes for cell motility* or to the *recruitment of ancient motile genes to new functions*. The loss of genes is a common aspect of intracellular bacteria and parasites (Moran and Wernegreen, 2000; Gil et al., 2004), since the stable environment provided by the host, and sometimes, the existence of secondary endosymbionts make redundant some genes (Pérez-Brocal et al., 2006). In endosymbionts, the loss of genes includes both those related to metabolic processes and those associated with the synthesis of the proteins of flagellar apparatus. As a result, their motility is completely lost. A representative example is provided by *Erwinia dacicola* (a prokaryotic symbiont of the Olive Fly *Bactrocera oleae*), which has a reduced number of genes for the amino acid and carbohydrate transport and metabolism, and a nearly complete loss of genes for cell motility compared to its free-living state (Estes, 2018).

Some endosymbionts, like *Buchnera aphidicola* (an endosymbiotic bacterium of pea aphids), keep their motile genes, but they cannot move, because the proteins expressed by their flagellar genes are supposed to be employed for protein transport functions, and not for motile functions (Maezawa et al., 2006). Flagellar genes are therefore used for a different purpose (likely protein transport), even though a potential pathogenic role cannot be excluded (Moya et al., 2008). As Toft and Fares (2008) pointed out, the endosymbiotic bacteria of insects usually lose their flagellar genes and they retain only the proteins of flagellum involved in protein export, whereas those involved in the synthesis of the hook and filament of flagella have generally been lost. Therefore, since the presence of flagella is unnecessary and energetically expensive, it has been suggested that the re-functionalization of the flagellar genes of endosymbionts (like in *B. aphidicola*) is the outcome of the adaptation of the symbiont to the intracellular niche of the host (Toft and Fares, 2008).

It has been shown that spirochaetes^9^ live on the surface – as ectosymbionts – of many protists (within the hindgut of termites) *without performing locomotion* (Iida et al., 2000; König et al., 2005). In spite of having flagella, spirochaetes cannot use them to move. However, the *unique* (so far known) example of bacterial ectosymbionts performing locomotion is represented by the spirochaetes living on *Mixotricha paradoxa* (a protist of the order of *Trichomonadida*) (Wenzel et al., 2003; König et al., 2005). *M. paradoxa* contains both endosymbionts (rod-like bacteria) and ectosymbionts (spirochaetes). Although *M. paradoxa* possesses four flagella^10^, its movement is performed by its spirochaetes. It has been proven that the loss of ectosymbionts or their inhibition by means of starvation or antibiotic treatment makes *M. paradoxa* unable to move (Radek and Nitsch, 2007). It is worth noting that many termite flagellates have been reported to have ectosymbionts with spirochaetes, but *only M. paradoxa* has spirochaetes that perform a coordinated movement in such a way that *M. paradoxa* can displace (Cleveland and Cleveland, 1966). The association of *M. paradoxa* and its ectosymbionts seems to be obligate not only for the movement but also for the performance of other vital functions of the symbiotic inter-identity (Radek and Nitsch, 2007). By contrast, the endosymbionts of *M. paradoxa*, as most of endosymbionts, cannot perform movement and are thought to perform a mitochondrion-like role.

The three symbiotic processes that we have so far examined reveal some important differences between them. In particular, biofilms use the motility of single bacteria for the primary attaching phase; then, when the EPS matrix begins to develop, the genes for motility are inhibited. During the breakdown of the EPS matrix, the genes for motility are re-activated and they allow single bacteria to get into the planktonic state. Endosymbiosis usually promotes the inhibition of symbiont motility especially through the loss or re-functionalization of genes for motility. Finally, ectosymbionts exhibit flagella that cannot move, except for the ectosymbiotic spirochaetes of *M. paradoxa*.

In general, in each of these three cases, the control of the motile interaction is a way to *contribute* to the *self-maintenance* of the overall symbiotic association. Indeed, the inhibition of motility of the bacteria of a biofilm keeps them in a stable position so as to favor the formation and the maintenance of the EPS matrix which in turn allows bacteria to interchange nutrients, metabolites, and to increase their immune response to pathogens and antibiotics. Likewise, the control of motility of endosymbionts and ectosymbionts indirectly affects the self-maintenance of the overall symbiotic association, because the loss of motile genes allows symbionts to spare ATP molecules that can be employed for performing physiological (notably metabolic) processes that are crucial for the whole association. Furthermore, the re-functionalization of motile genes allows symbionts to perform important mechanisms (e.g., protein transport) that improve the metabolic relationships between the symbiont and the host. Finally, the spirochaetes of *M. paradoxa* make a direct contribution to the motility of the overall symbiotic association and as such enable it to reach its nutrients and to autonomously interact with its surroundings.

A particular theoretical interest is aroused by endosymbionts, as this form of symbiosis is considered as the root of eukaryogenesis, notably of mitochondria and plastids (Margulis, 1967). We may therefore suppose that the inhibition of motility, which plays a cardinal role in endosymbionts, should be also an important feature for understanding the transition from the endosymbiotic to the organelle form of mitochondria and plastids.

## Mobility of Eukaryotic Organelles

Both mitochondria and plastids exhibit extremely reduced genomes and can synthesize few proteins involved in the electron transport chain and F_0_F_1_ATPase (mitochondria) or in the photosynthetic apparatus and in the transcription/translation apparatus (plastids). Thus, they lack almost all the genes (of prokaryotic origin) for the most fundamental cellular physiological functions, including those for flagella. Although neither mitochondria nor plastids can *spontaneously move*, they *are instead moved* by the *eukaryotic cytoskeleton*. Since the motility of mitochondria and plastids is *hetero-driven* by cytoskeletal filaments and not self-driven by the organelle itself, they exhibit *mobility* and *not motility*. By the former we mean the movement of an entity performed by another entity; whereas the latter is the motion performed by the entity itself.

Mitochondria and plastids *are moved* by two main cytoskeletal filaments: *microtubules* and *microfilaments*^11^. The former are composed of *polymers of tubulin* that are responsible not only for cell motility, but also for several cellular functions, such as the transport of chromosomes during cell division, the maintenance of cell shape, the transport of intracellular materials, and the movement of cell membrane components. The latter are *filaments of actin* that control cell motility and cell separation (cytokinesis). Microfilaments can generate movement in two ways: by a sliding movement of actin and myosin filaments against each other or assembling and disassembling the microfilament bundles. In the former case, when myosin heads bind ATP molecules, they have a high affinity for actin and this drives the bond between actin and myosin. The hydrolysis of ATP allows myosin heads to slightly rotate and to become disengaged from myosin^12^. In the latter case, actin filaments polymerize and depolymerize so as to produce motion.

Mitochondria use cytoskeletal proteins as tracks for their *directional* (anterograde or retrograde) *movement* by means of a coordinated action between microtubules and microfilaments (Anesti and Scorrano, 2006). Both microtubules and microfilaments are important for mitochondrial movement and contribute to mitochondrial displacement in a different way. A protein (the mitochondria-microtubule binder protein, mmb1p) seems to be responsible for the bond between *mitochondria and microtubules* (Fu et al., 2011), giving rise to a functional interdependence between them. Indeed, on the one hand, mitochondria reduce microtubule shrinkage rate and contribute to the stabilization of microtubules; on the other, they are controlled by microtubules, because microtubules are *scaffolds* to maintain the position of mitochondria (Pon, 2011). Furthermore, the bond between *mitochondria and actin cables*, mediated by the mitochore complex, drives mitochondrial movement both in an *anterograde and a retrograde direction*. The anterograde movement of mitochondria is driven by the Arp2/3 complex^13^ that stimulates actin polymerization for the generation of anterograde force (Boldogh and Pon, 2006; Wu et al., 2013). Finally, intermediate filaments maintain cell shape by bearing tension, whereas microtubules resist compression (Wu et al., 2013). The movement of mitochondria along actin and tubulin is made possible by molecular motors (myosin binds to actin, whereas dynein and kinesin bind to tubulin), which are proteins powered by ATP hydrolysis and consisting of three main parts: the *head domain* binding the cytoskeletal filament, the *neck domain* acting as a lever arm for transducing chemical energy into mechanical energy, and the *tail domain* binding the cargo. Molecular motors bind organelles at the tail domain and cytoskeletal filaments at the head domain in such a way as to act as a “cart” for the movement of organelles.

The movement of chloroplasts is mainly due to actin filaments which are localized at the interface between the chloroplast and the plasma membrane. In particular, *motor proteins* and the *polymerization of actin filaments* are the main actors of chloroplast movement. The motor proteins responsible for plastid movement are different from those involved in mitochondrial movement (i.e., myosin, dynein, and kinesin) and are based on the *actomyosin system* (Shimmen and Yokota, 2004). Actin polymerization is induced by environmental stimuli (e.g., changes in light intensity or mechanical touch) and controlled by a number of mechanisms not yet clearly understood. It is believed that the protein CHUP1^14^ may play a major role, because it binds to profilin which supports actin assembly (Wada and Kong, 2018). The polymerization of chloroplast-actin filaments is considered the most likely candidate mechanism to generate the force required for chloroplast movement (Wada and Kong, 2018). Microtubules of plant cells are thought to contribute to chloroplast movement inasmuch as they support the functioning of actin filaments (Brandizzi and Wasteneys, 2013).

Both *mitochondrial and plastid movement* make a substantial contribution to the physiology of the eukaryotic cell, insofar as mitochondria and plastids can be more spatially close to the other eukaryotic organelles and hence favor *intercellular communication*.

Cytoskeletal-driven movement is intimately connected with the so-called “mitochondrial dynamics” consisting of cycles of fusion and division, as the disassembly of microtubules eliminates mitochondrial motility and, as a result, makes possible fusion and fission events (Bartolák-Suki et al., 2017). Fusion and fission events involve changes both in mitochondrial shape and in mitochondrial membranes, inasmuch as fusion entails the merger of mitochondrial membranes, whereas fission needs the formation of a septum within the membrane, leading to daughter mitochondria. Fusion and fission play a pivotal role in several eukaryotic cellular processes, insofar as they are involved in the maintenance of calcium homeostasis (through the connection with endoplasmic reticulum), cell development and cellular division. Furthermore, mitochondrial dynamics are involved in *cell survival processes*, including autophagy, apoptosis, and necroptosis (Xie et al., 2018). The mobility of mitochondria involves not only their fusion and fission but also their capacity to interact with other eukaryotic organelles *via signaling pathways* in such a way as to regulate many cellular functions. More particularly, mitochondria interact with endoplasmic reticulum, peroxisomes, lysosomes and Golgi apparatus^15^.

In plants, the movement of chloroplasts is important for plant growth and development. Depending on light intensity, plastids can distribute differently in the plant cells (randomly in bundle sheath cells, centripetally in the vascular tissue, and centrifugally around the periphery of the bundle sheath cells) so as to favor the exchange of metabolites. Both cytoplasmic ATP levels and CO_2_ diffusion are important physiological factors affecting chloroplast movement and positioning (Takagi et al., 2009). Moreover, the spatial proximity of plastids to the plasma membrane permits the maximization of the transport of CO_2_ from the intercellular airspace to the site of CO_2_ fixation (the chloroplast stroma), and therefore, makes photosynthesis more efficient (Takagi et al., 2009).

In spite of playing a different role in the control of the movement of chloroplasts and mitochondria, both actin filaments and microtubules make a significant contribution to the positioning of the organelles within the eukaryotic cell in such a way that *intracellular communication* and other important *physiological cellular functions* can be performed. The controlled motion of organelles occupies a crucial organizational role that, on the one hand, makes a dramatic difference with symbiotic association, and, on the other, suggests the critical importance of the *cytoskeleton* in the *transition* from prokaryotic to eukaryotic cell.

## Interactive Dynamics and the Organizational Role of the Eukaryotic Cytoskeleton

The previous two sections have examined the motility of symbionts and organelles, focusing on their different functional contributions to the eukaryotic cell. In both cases the control of the motility of the parts is aimed at satisfying physiological requirements of the eukaryotic cell. However, ongoing endosymbionts and organelles of endosymbiotic origin exhibit a different control of motile capacities which can be understood partly by exploring the evolutionary innovations introduced by the eukaryotic cytoskeleton (compared to the prokaryotic one), partly by analyzing the different roles played by endosymbionts and organelles within the eukaryotic cell.

Despite the discovery of bacterial homologs of actin (Bork et al., 1992), tubulin (de Boer et al., 1992; RayChaudhuri and Park, 1992; Mukherjee et al., 1993) and intermediate filaments (Margolin, 2004)^16^, the eukaryotic cytoskeleton performs new functions, not present in the prokaryotic cell, that allow eukaryotes to move organelles or bacterial pathogens within themselves. Compared to the prokaryotic cytoskeleton, which is involved in the production of cell wall, the maintenance of cell shape and the support for cell division, the eukaryotic one performs several different functions, including *intracellular transport of organelles* and *intracellular signaling*. Intracellular transport is unique to the eukaryotic cell^17^, because organelles are enclosed in membranes requiring vesicles for transporting intracellular cargos (Bonifacino and Glick, 2004). Intracellular transport is performed by *molecular machines* that transport cargoes along actin filaments (myosin) or microtubules (dynein and kinesin) by exploiting ATP hydrolysis (Dawson and Paredez, 2013; Jékely, 2014). The force^18^ generated by the eukaryotic cytoskeleton permits a new kind of spatial organization within the eukaryotic cell that cannot be found in the prokaryotic one.

The *remodeling of filamentous actin* plays a pivotal role both in cell motility (Diez et al., 2005) and is triggered by a variety of cellular signals, including PIP_2_ ^19^, Ca^2+^, and small GTPases (Takenawa and Itoh, 2001). The stimulation of purinergic receptors, due to the rise of Ca^2+^, allows actin filaments to accumulate around intracellular organelles in such a way as to slow down their movement through the cytoplasm. The major nucleators of actin polymerization are the Arp2/3 complex and the members of the formin family, which give rise to different actin structures: the Arp 2/3 complex produces branched filaments, whereas formin straight and bundled filaments (Diez et al., 2005).

Since both the endosymbionts (of protists and insects) and organelles are embedded in eukaryotic cells having a eukaryotic cytoskeleton, both should be moved and displaced by molecular motors along actin filaments and microtubules. Nevertheless, the fact that only organelles, and not also endosymbionts, have a cytoskeleton-driven movement is closely connected with the different functional role that organelles and endosymbionts play within the eukaryotic cell.

The movement of organelles permits intracellular communication *via* vesicle-mediated pathways^20^: the interchange of molecules (e.g., ions, proteins, lipids, etc.) among mitochondria (and plastids), endoplasmic reticulum, Golgi apparatus, lysosomes, and nucleus would not occur if these organelles were not be *spatially close* (Perico and Sparkes, 2018). In turn, the delivery and the coordinated transfer of molecules enable organelles to perform important physiological tasks that collectively contribute to the self-maintenance of the eukaryotic cell. For example, the spatial proximity between endoplasmic reticulum and Golgi apparatus allows the movement of proteins between them as well as the closeness between mitochondria and other organelles favors the interchange of reducing equivalents and ATP molecules. Since organelle movement plays such a crucial role, the eukaryotic cell *modulates* the distribution of the organelles with *spatiotemporal accuracy* by means of changes in network and motor properties (e.g., polarization, signaling, motor mobility, etc.) (Ando et al., 2015; van Bergeijk et al., 2015).

Unlike organelles, endosymbionts do not perform regulatory and homeostatic mechanisms for the host. Accordingly, they require neither displacement nor a fine-tuned dynamic spatiotemporal control from the eukaryotic cell. Indeed, endosymbionts usually provide the host with enzymes necessary for performing catabolic or anabolic pathways (e.g., the enzymes for amino acid anabolism of sap-feeding insects), which are absent or incomplete in the host. The enzymes synthesized by endosymbionts are targeted to the plasma membrane of the host through co-translation or post-translation pathway without the need for spatial proximity to the membrane contact sites of eukaryotic organelles. For these reasons, the host does not need to consume energy to displace endosymbionts and they can be kept in an extremely stable position during the symbiotic association. It is worthy of note that the eukaryotic cytoskeleton can be also employed by bacterial pathogens for performing invasion strategies (Haglund and Welch, 2011; Gouin et al., 2015) by exploiting actin polymerization. Therefore, the fact that (bacterial) endosymbionts are not moved by the cytoskeleton is likely not due to a cytoskeletal limitation, but rather to the uselessness of this displacement within the eukaryotic context.

The eukaryotic cytoskeleton is a fundamental step not only in the transition from prokaryotic to eukaryotic cell but also in the evolution of mitochondria and plastids from long-term stable endosymbionts to organelles. The eukaryotic cytoskeleton has given rise to an extremely dynamic and interconnected network within the eukaryotic cell that has led to complex forms of *communication* and a fine-tuned *spatiotemporal localization* of eukaryotic organelles in such a way that the degree of cohesion and mutual dependence among the parts considerably increased. This was a very important innovation during eukaryogenesis because it opened up a more sophisticated form of *intracellular communication* (vesicular transport instead of simple diffusion) and an *effective control over the positioning of organelles*. These important biological novelties have made an important contribution to the overall functional integration of the eukaryotic cell.

Special attention should be paid to the major contribution made by the eukaryotic cytoskeleton to the transition from endosymbiotic proto-mitochondria and proto-plastids to organelles. Both mitochondria and plastids have an endosymbiotic origin (α-proteobacteria were likely the ancestors of mitochondria, whereas cyanobacteria of plastids) and they transformed into organelles over millions of years (Martin et al., 2015). It has been stressed that the main events that allowed endosymbionts to become organelles were the massive transfer of genes to the eukaryotic nucleus (endosymbiotic gene transfer) and the appearance of protein import machineries in the membranes of proto-mitochondria and proto-plastids (Theissen and Martin, 2006). We hypothesize that at some point in eukaryogenesis the eukaryotic cytoskeleton must have played a pivotal role in the transformation of proto-mitochondria and proto-plastids into organelles.

Indeed, given that mitochondria and plastids were endosymbionts, they lost most of their genes, including those for cell motility. It is therefore likely that in an initial phase of eukaryogenesis mitochondria and plastids were immobile or, at least, with a very reduced ability to move. Yet, since proto-mitochondria and proto-plastids were progressively performing regulatory and homeostatic mechanisms, it was necessary to provide some mechanisms for displacing and putting them close to other eukaryotic organelles in order to ensure intracellular communication. From this perspective, the eukaryotic cytoskeleton is no longer just a bunch of filaments for controlling cell shape, but an extremely dynamic structure that has allowed mitochondria, plastids, and the other eukaryotic organelles to achieve a high degree of functional integration.

## Concluding Remarks: The Relationship Between Motility and Biological Autonomy

In the light of the theoretical results achieved in the previous sections, we shall explore in this concluding section how the control of the motility of the individual parts affects their *interactive autonomy* (i.e., agency) and the *constitutive autonomy* of the whole collective organization.

The inhibition of motility is a biological phenomenon that both symbionts (except for the ectosymbionts of *M. paradoxa*) and organelles have in common. Nevertheless, we have shown that the eukaryotic cytoskeleton provides organelles with a mobility which is completely controlled by the eukaryotic cell. In the light of the distinction between mobility and motility (see section “Mobility of Eukaryotic Organelles”), it is therefore clear that the notion of “motility” implies the concept of “agency,” inasmuch as the *autonomous movement* is a way to interact and functionally modify the surroundings. Since both symbionts and organelles have lost their motile capacities or, if they are present, they are driven by the eukaryotic cell, is it possible to consider (endo)symbionts and organelles genuine agents?

In order to address this question, let us consider what a minimal agent is and then evaluate whether or not symbionts and organelles satisfy the conditions for minimal agency. A definition of minimal agency has recently been provided by Moreno (2018), who has stressed that a minimal agent is a system detecting relevant features of the surroundings (e.g., nutrients) and triggering processes that can functionally modify the environmental conditions. The effector mechanisms must be controlled *from within* by means of a *self-production network* (i.e., metabolism) and *a regulatory system* that is dynamically decoupled from the self-production network (Moreno, 2018, p. 295).

The bacteria forming a biofilm and attaching to the biotic surface of a multicellular eukaryote are able to detect environmental signals and nutrients which are present in the surface and to perform effector mechanisms that modify their host. For example, bacteria constituting the biofilm of dental plaque can detect environmental signals such as pH or the nutrients (amino acids, proteins, glycoproteins) provided by saliva and gingival fluid and they release enzymes that produce infectious diseases (like caries or periodontitis) or inflammatory states (like gingivitis) in the host. The release of enzymes of biofilms is tightly controlled by the QS system of biofilms. Likewise, endosymbionts detect the nutrients released by their host in the host cytoplasm and they synthesize and release enzymes for metabolic pathways (e.g., the enzymes for amino acid synthesis). The production of enzymes is controlled by the genes of the endosymbiont, not by the host. Ectosymbionts (like the spirochaetes of *M. paradoxa.*) detect environmental signals that activate their flagella which in turn allow *M. paradoxa* to move. The regulation of the movement of spirochaetes is made by the symbiont and not by the host. In each of these three cases, even though motility can be inhibited or lost (in bacteria of biofilms or in endosymbionts), the symbionts still preserve their ability to *autonomously* interact with their host and the interactive processes are controlled *from within* and not by the host. For this reason, they can be considered as genuine agents, even if in nested hierarchical organizations of symbionts “many functions of the individuated parts are transferred to the higher collective level. These facts often lead to an ultra-simplification of certain agents (e.g., endosymbionts)” (Moreno, 2018, p. 306).

Organelles exhibit a pretty different organization. They perform a wide variety of functions that go far beyond metabolic contributions (like in endosymbionts) and that include regulatory and homeostatic mechanisms of the eukaryotic cell. As such, their effector mechanisms functionally change their surroundings (i.e., the eukaryotic cell) by controlling the eukaryotic cell as a whole. A clear example is provided by mitochondrial dynamics (fusion and fission) which collectively control pivotal events of the eukaryotic cell, such as apoptosis, autophagy, cell development, etc. Furthermore, the mobility of organelles, fulfilled by the cytoskeleton, allows them to efficiently communicate among each other in such a way as to perform pivotal physiological processes. Apparently, the organelles of endosymbiotic origin seem genuine agents within a “macro-agent” represented by the eukaryotic cell. However, since almost all of their genes have been transferred to the eukaryotic nucleus, the proteins controlling their functions are genetically expressed and controlled by the eukaryotic nucleus^21^. Accordingly, given that the regulation of their effector mechanisms is placed *outside the organelle*, and *not within*, they *cannot be considered genuine agents*. For example, the key proteins regulating mitochondrial fusion (Mtf1 and Mtf2, and OPA1) and fission (Drp1, Fis1, and DnmP1), in spite of being placed within the outer and inner mitochondrial membrane, are *expressed* and *genetically controlled* by the genes placed in the eukaryotic nucleus. The endosymbiotic gene transfer and the genetic control and expression made by the eukaryotic nucleus represent the dividing line between organelles of endosymbiotic origin and ongoing endosymbionts.

In line with the definition of “minimal agency” provided by Moreno (2018), we think that what defines a minimal agent is the ability of functionally modifying its surroundings by virtue of some effector mechanisms that are controlled from within. If we accept this characterization of minimal agents, symbionts can be considered agents, even though they do not exhibit the coupling between sensory inputs and motor outputs. Sensorimotor coupling is an important aspect of agency in prokaryotic and eukaryotic forms of life (Moreno and Etxeberria, 2005; Moreno and Mossio, 2015; Di Paolo et al., 2017); however, it fails to explain why symbionts can be considered agents and why mitochondria and plastids cannot. Moreover, it is worth emphasizing that the acknowledgement of symbionts as genuine agents allows a better characterization of the biological status of symbiotic associations. Indeed, the identity of a symbiotic association relies on the kind of interactions (metabolic, immunological, developmental, etc.) among symbiotic partners. The *control of the motility* of the symbiont plays a very important role in the emergence of a *collective inter-identity*, insofar as it weakens the interactive capacities of the symbionts –without completely undermining them- to the benefit of the constitutive processes (metabolism, regulatory mechanisms, development, etc.) of the symbiotic association as a whole.

Considering symbionts as real agents is extremely important not only for explaining the emergence of collective inter-identities, but also for clarifying the difference between endosymbionts and organelles of endosymbiotic origin. The ultimate outcome of the transition from the former to the latter was the *loss* of *autonomy* and, therefore, *agential capacities*. This can be mostly attributed to the transference of genes to the host and the subsequent control of their functions by the eukaryotic cell. The reason why mitochondria and plastids are *not* agents is based on the fact that they are genetically controlled by the eukaryotic nucleus. Certainly, they perform functions that change the eukaryotic cell and exhibit motor capacities driven by cytoskeleton, but the absence of an *internal* regulation of these processes do not make them agents. The interactive capacities of mitochondria and plastids can be likened to the footballers of a table football: they “kick” the little ball and they perform an action which modifies the position of the little ball; however, their movement is completely controlled by a human being who decides *when* and *how* a footballer moves so as to push the little ball toward the goal area of the opponent.

It is important to stress that, even though a biological system has lost its *autonomous* interactive capacities, this does not necessarily imply the complete loss of interactive capacities. The case of the organelles of endosymbiotic origin is extremely clear in this respect: organelles have lost their autonomy and their agential abilities because of a massive endosymbiotic gene transfer that has placed their genetic control in the eukaryotic nucleus. However, mitochondria and plastids communicate with the other eukaryotic organelles by means of vesicle-mediated pathways and thanks to cytoskeletal proteins. This *communication* is a kind of *interaction* that does not involve agential abilities, precisely because it is a functional modification of the environment without an internal control.

We have so far discussed the relationship between agency and interactive capacities in symbionts and organelles. We are now able to provide an answer to the key question of this paper: how is the *motility of individual parts* related to the *constitutive dimension of a collective identity*? The answer lies in the fact that the control of the motility of the part is aimed at maintaining the collective identity as a whole by constraining a flux of energy and matter and, as such, it keeps the nested organization far from thermodynamic equilibrium (Mossio and Moreno, 2010; Moreno and Mossio, 2015). Both the loss or inhibition of motility (in symbionts) and the cytoskeleton-driven mobility (in organelles) are ways to contribute to the self-maintenance of the nested organization, inasmuch as they are a fundamental support for the maintenance of other pivotal interactions (e.g., the metabolic fluxes between the part and the whole, the intracellular communication among organelles, etc.) which collectively sustain a nested organization as a whole.

## Author Contributions

GM has designed the research work and written all the sections of the article. The author confirms being the sole contributor of this work and has approved it for publication.

### Conflict of Interest Statement

The author declares that the research was conducted in the absence of any commercial or financial relationships that could be construed as a potential conflict of interest.

## Footnotes

^1^In this article, we explore the relationship between motility and self-maintenance by employing some expressions (“so as to,” “in order to,” etc.) that can suggest a teleological meaning. However, all these “teleological” expressions should be understood within the organizational framework for biological functions, according to which biological functions (including motile capacities and sensorimotor abilities) are aimed at self-maintaining a biological organization within a regime of organizational closure (see, for example, Moreno and Mossio, 2015, chap. 3; Mossio and Bich, 2017).

^2^A clear example of sensorimotor coupling is bacterial chemotaxis (e.g. in *E. coli*), since the detection of attractants or repellents in the environment triggers a signaling cascade that modifies the frequency and the direction of the motile system (i.e. flagella).

^3^As pointed out by Moreno and Etxeberria (2005) and Barandiaran and Moreno (2009), motility and behavioral agency are strongly affected by the size of the organism, because the increase in size makes more difficult not only the correlation between sensor and effector surfaces “because of the slow velocity of diffusion processes” (Moreno and Mossio, 2015, p. 103), but also the achievement of a bodily coordination for displacement.

^4^Although biofilms are a kind of symbiotic association that can live independently from an eukaryotic host (indeed, biofilms can attach to abiotic surfaces), they usually attach to biotic surfaces provided by a (multicellular) eukaryotic host. Accordingly, we think that biofilms can be considered as a specific kind of transient symbiont (i.e., a parasite) of eukaryotic cells and, therefore, it is useful to evaluate the constraints posed on the motility of the bacterial components by the extracellular polymeric matrix and how this affects the relationship with the eukaryotic host.

^5^The EPS matrix is a three-dimensional organization that keeps bacteria very close to one another so as to increase the cohesiveness and coordination of component bacteria, compared to their planktonic state. The EPS matrix enables a biofilm to exhibit a strong metabolic codependence and synthrophy, common developmental dynamics, and an enhanced immune response of the individual bacteria.

^6^One of the most relevant post-translational modifications is the bond between the second-messenger c-di-GMP and the PilZ domain in the *ycgR* gene (Hengge, 2009; Ko and Park, 2009).

^7^A number of transcriptional regulatory mechanisms may either activate (e.g., Rcs system and CsrA) or inhibit (e.g., FliZ and CsgD) the expression of flagellar genes in such a way that motility gene expression appears to be strongly controlled during the transition from motile to sessile state of bacteria.

^8^QS system plays an important role in the inhibition of chemotaxis and motion of bacteria. For example, the autoinducer 2 (AI-2) determines a cascade of events that dephosphorylate the response regulator CheY, leading to a counter-clockwise rotation of flagella and smooth swimming (Blat and Eisenbach, 1994).

^9^Spirochaetes are bacteria with spiral shapes.

^10^The flagella of *M. paradoxa* seem to be an ancient relic rather than a functional part of the protist.

^11^A third system, which can be found in the eukaryotic cells of vertebrates and some invertebrates, is represented by the intermediate filaments which contribute to the maintenance of cell-shape.

^12^In muscle cells the sliding movement is mediated by tropomyosin and troponin, which bind to the actin filament (Cappucinelli, 1980).

^13^The Arp2/3 is a protein complex that regulates the polymerization and depolymerization of actin filaments.

^14^CHUP1 stands for Chloroplast Unusual Positioning 1.

^15^Lysosomes play an important role in amino acid sensing, exocytosis, plasma membrane repair, transcriptional regulation and also acts as reservoir of amino acids, metabolites and ions. Endoplasmic reticulum is relevant for protein folding, Ca^2+^ storage, and metabolism of carbohydrates and lipids. Peroxisomes perform the β-oxidation of fatty acids (Diogo et al., 2018).

^16^Homologs proteins for actin are FtsA, MreB, MamK, ParM and Alf; for tubulin are FtsZ, TubZ, PhuZ, and BtubA/B; and for intermediate filaments the crescentin protein (Pilhofer and Jensen, 2013).

^17^Prokaryotes interchange cargos by means of simple diffusion.

^18^The main mechanisms underlying the generation of cytoskeletal force include *filament growth*, *filament shrinkage*, and *molecular motors walking on filaments* (Jékely, 2014).

^19^PIP_2_ stands for phosphatidylinositol 4,5-bisphosphate, which is a phospholipid involved in the organization and polymerization of filamentous actin by binding to F-actin regulatory proteins.

^20^The interaction occurs at the membrane contact sites (MCSs) which are zones of apposition between two organelles.

^21^An exception is represented by those few genes already present in mitochondrial and chloroplast genomes which control oxidative metabolism (in mitochondria) and photosynthesis (in chloroplasts).
